# Synthesis, crystal structure and thermal properties of poly[di-μ-bromido-(μ-2,5-di­methyl­pyrazine)cadmium(II)]

**DOI:** 10.1107/S2056989024011824

**Published:** 2025-01-01

**Authors:** Christian Näther

**Affiliations:** a Institut für Anorganische Chemie, Universität Kiel, Max-Eyth.-Str. 2, 24118 Kiel, Germany; University of Aberdeen, United Kingdom

**Keywords:** synthesis, thermal properties, cadmium bromide, coordination polymer, 2,5-di­methyl­pyrazine, crystal structure

## Abstract

The title compound, [CdBr_2_(C_6_H_8_N_2_)]_*n*_, was prepared by the reaction of cadmium bromide with 2,5-di­methyl­pyrazine in water. Its asymmetric unit consists of one Cd cation and one 2,5-di­methyl­pyrazine ligand that are located on a crystallographic mirror plane as well as one bromide anion that occupies a general position. The Cd cations are sixfold coordinated by four bromide anions and two 2,5-di­methyl­pyrazine ligands within slightly distorted *trans*-CdBr_4_N_2_ octa­hedra. The cations are linked into [100] chains *via* pairs of bridging bromide anions that are further connected into (001) layers by the bridging 2,5-di­methyl­pyrazine ligands. Powder X-ray diffraction (PXRD) shows that a pure crystalline phase has been obtained. Thermogravimetry coupled to differential thermoanalysis (TG-TDA) reveal that the 2,5-di­methyl­pyrazine ligands are removed in two separate steps leading to the formation of a compound with the composition (CdBr_2_)_2_(2,5-di­methyl­pyrazine) that decomposes into CdBr_2_ upon further heating. PXRD measurements of the residue obtained after the first mass loss show that a new crystalline phase has been formed.

## Abstract 2

In the title compound, the cadmium cations are octa­hedrally coordinated by four bromide anions and two 2–5-di­methyl­pyrazine ligands and linked into chains *via* pairs of μ-1,1-bridging Br^−^ anions that are further connected into layers by the bridging 2,5-di­methyl­pyrazine coligands.

## 1. Chemical context

For several years, we and others have been inter­ested in the synthesis and crystal structures of coordination compounds based on transition metal halides and neutral organic coligands. In the beginning, we focused on compounds based on Cu^I^, because they show an extremely versatile structural behavior (Kromp & Sheldrick, 1999 ▸; Peng *et al.*, 2010 ▸; Näther & Jess, 2002 ▸, 2004 ▸; Li *et al.*, 2005 ▸). Such compounds usually consist of Cu*X* subunits that can comprise monomeric and dimeric units but also different kinds of chains that are further linked into more condensed networks when bridging coligands are used in the synthesis. In the course of our systematic work we found that upon heating, such compounds frequently lose their coligands in a stepwise manner, which leads to the formation of new copper halide compounds as inter­mediates that consist of condensed Cu*X* networks (Näther *et al.*, 2001 ▸, 2002 ▸). More recently, we have shown that this synthetic route can also be expanded to coordination compounds with, *e.g.* Zn*X*_2_ and Cd*X*_2_ (*X* = Cl, Br, I), even if they, with few exceptions (Näther *et al.*, 2007 ▸), do not show the same structural variability as the Cu compounds.

In the course of our project we especially used bridging coligands such as pyrazine derivatives to prepare compounds with more condensed networks. Some compounds with Cd*X*_2_ (*X* = Cl, Br, I) and pyrazine (C_4_H_4_N_2_) have already been reported, including Cd*X*_2_(pyrazine) [*X* = Cl, Br, I, Cambridge Structural Database refcodes TISSUJ (Pickardt & Staub, 1996 ▸), RINSIQ and RINSOW (Bailey & Pennington, 1997 ▸); RINSOW01 and RINSIQ01 (Pickardt & Staub, 1997 ▸)], in which the Cd cations are linked by pairs of bridging halide anions into chains, which are further connected into layers by the pyrazine coligands. In this context we have reported on Cd*X*_2_ compounds with 2-chloro and 2-methyl­pyrazine, for which a different thermal reactivity was observed (Näther *et al.*, 2017 ▸). In the coligand-rich compounds Cd*X*_2_(*L*)_2_ (*X* = Cl, Br, I, *L* = 2-chloro and methyl­pyrazine: QAWHOO, QAWGON, QAWGUT, QAWHAA, QAWHEE and QAWHII; Näther *et al.*, 2017 ▸), the Cd cations are octa­hedrally coordinated and linked into chains by pairs of bridging halide anions. If the 2-methyl­pyrazine compounds are heated, a transformation into 2-methyl­pyrazine-deficient compounds with the composition Cd*X*_2_(2-methyl­pyrazine) (*X* = Cl, Br, I) is observed, in which the Cd*X*_2_ chains are further linked into layers as with the pyrazine compounds mentioned above. In contrast, for the 2-chloro­pyrazine compounds, no 2-chloro­pyrazine-deficient compounds can be obtained and they can also not be prepared from solution (Näther *et al.*, 2017 ▸).
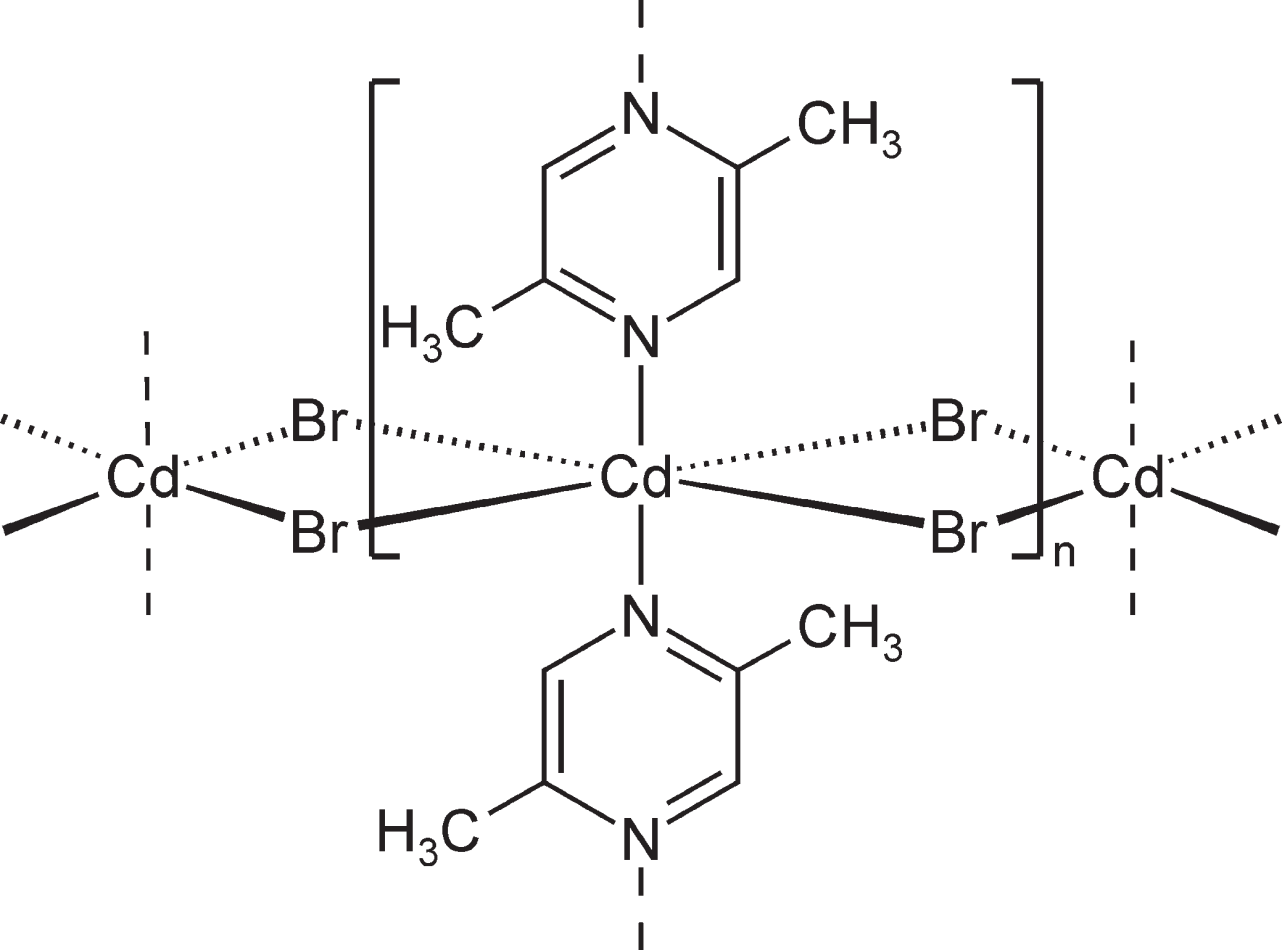


In a continuation of this work we became inter­ested in compounds with 2,5-di­methyl­pyrazine (C_6_H_8_N_2_) in which the coordination to each of the N atoms is sterically hindered because of the bulky methyl groups. A compound with the composition CdI_2_(2,5-di­methyl­pyrazine) is already reported in the CSD (EHEQUG; Rogers, 2020 ▸). Surprisingly, the structure of this compound is completely different from that of the 2-methyl and 2-chloro­pyrazine compounds mentioned above. In EHEQUG, the Cd cations are tetra­hedrally coordinated by two iodide anions and two 2,5-di­methyl­pyrazine coligands and linked into chains by the coligands. Compounds with CdCl_2_ or CdBr_2_ and 2,5-di­methyl­pyrazine have not been reported. In the course of our investigations, we obtained crystals of the title compound, (**I**), by the reaction of CdBr_2_ and 2,5-di­methyl­pyrazine, which were characterized by single crystal X-ray diffraction.

## 2. Structural commentary

**Figure 1:**
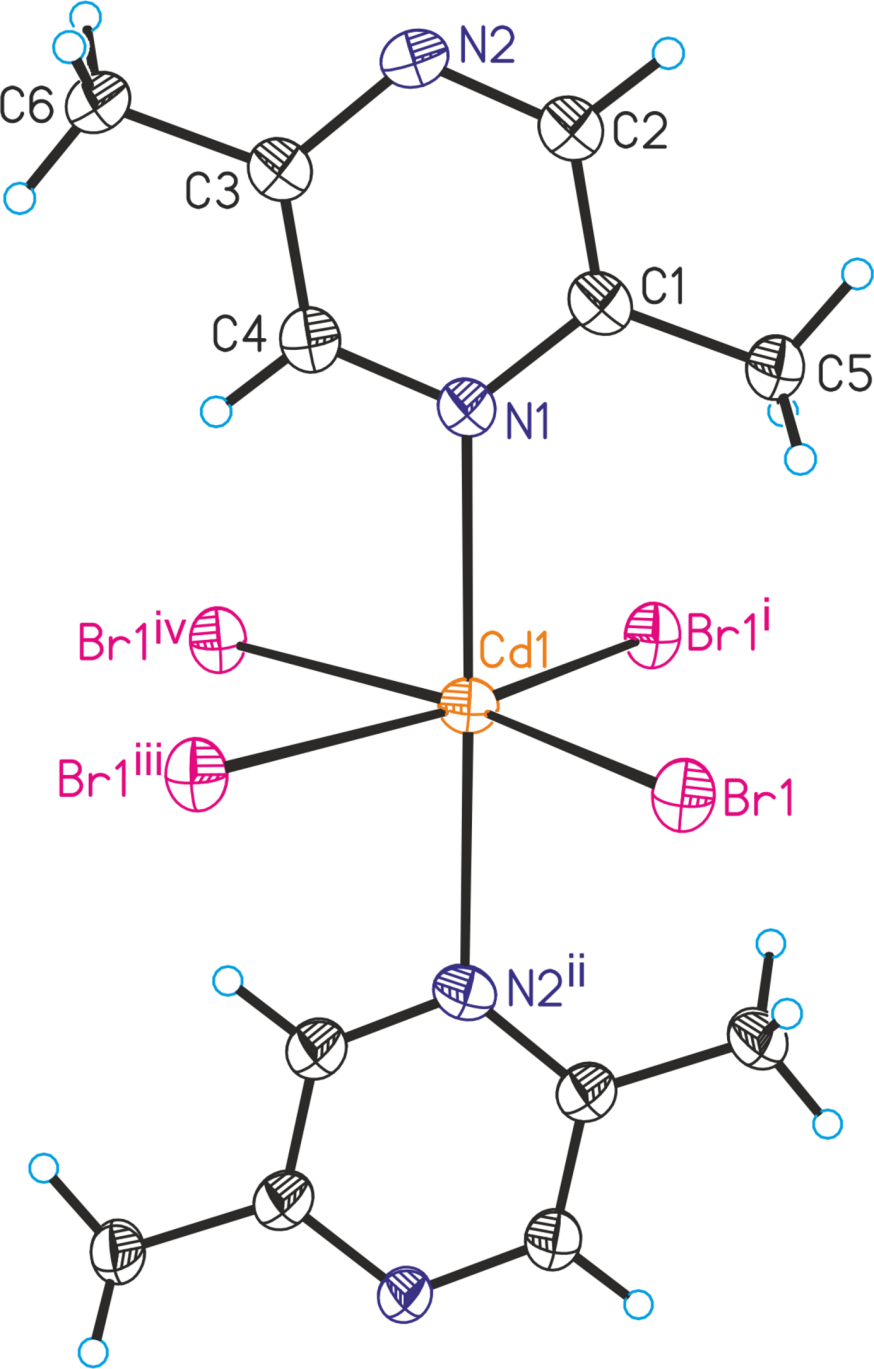
The crystal structure of (**I**) with labeling and displacement ellipsoids drawn at the 50% probability level. Symmetry codes for the generation of equivalent atoms: (i) −*x* + 1, *y*, *z*; (ii) *x*, *y* + 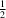, −*z* + 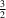; (iii) −*x* + 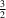, *y*, −*z* + 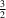; (iv) *x* − 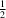, *y*, −*z* + 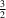; (v) *x* + 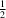, *y*, −*z* + 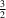.

**Figure 2:**
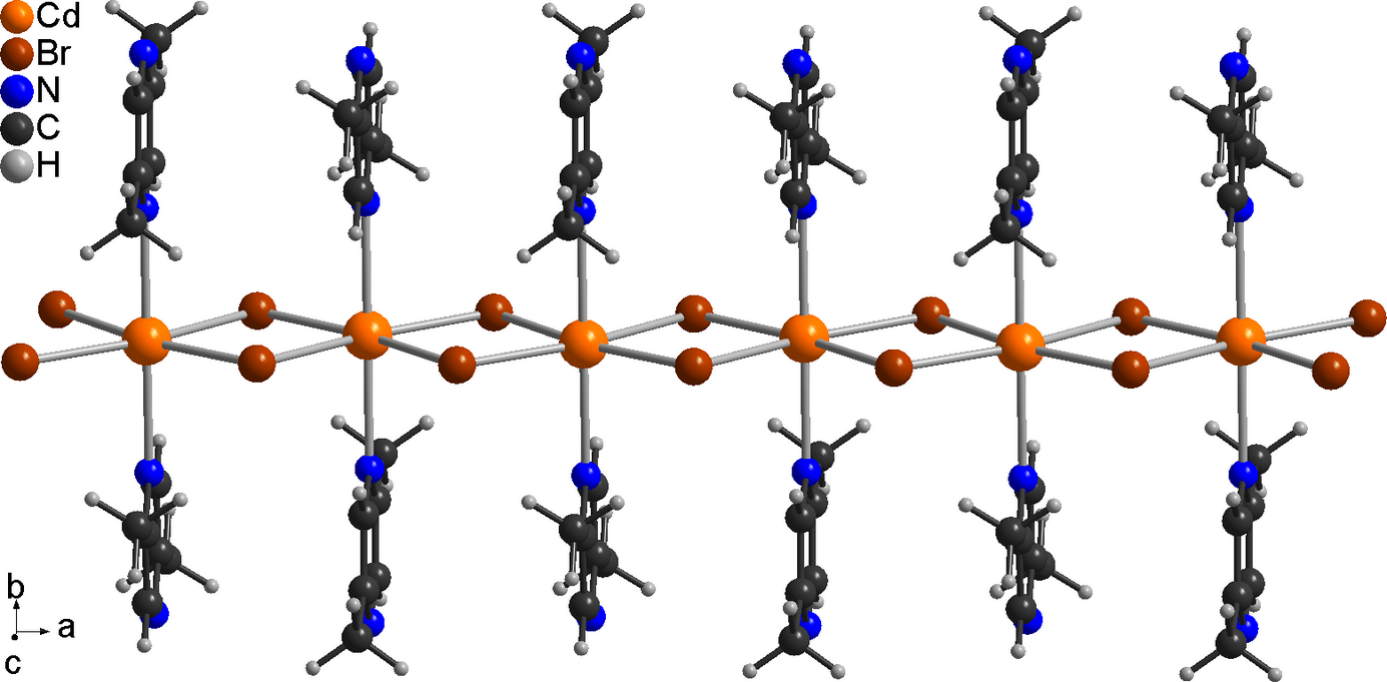
Crystal structure of (**I**) with view of part of one [100] chain.

**Figure 3:**
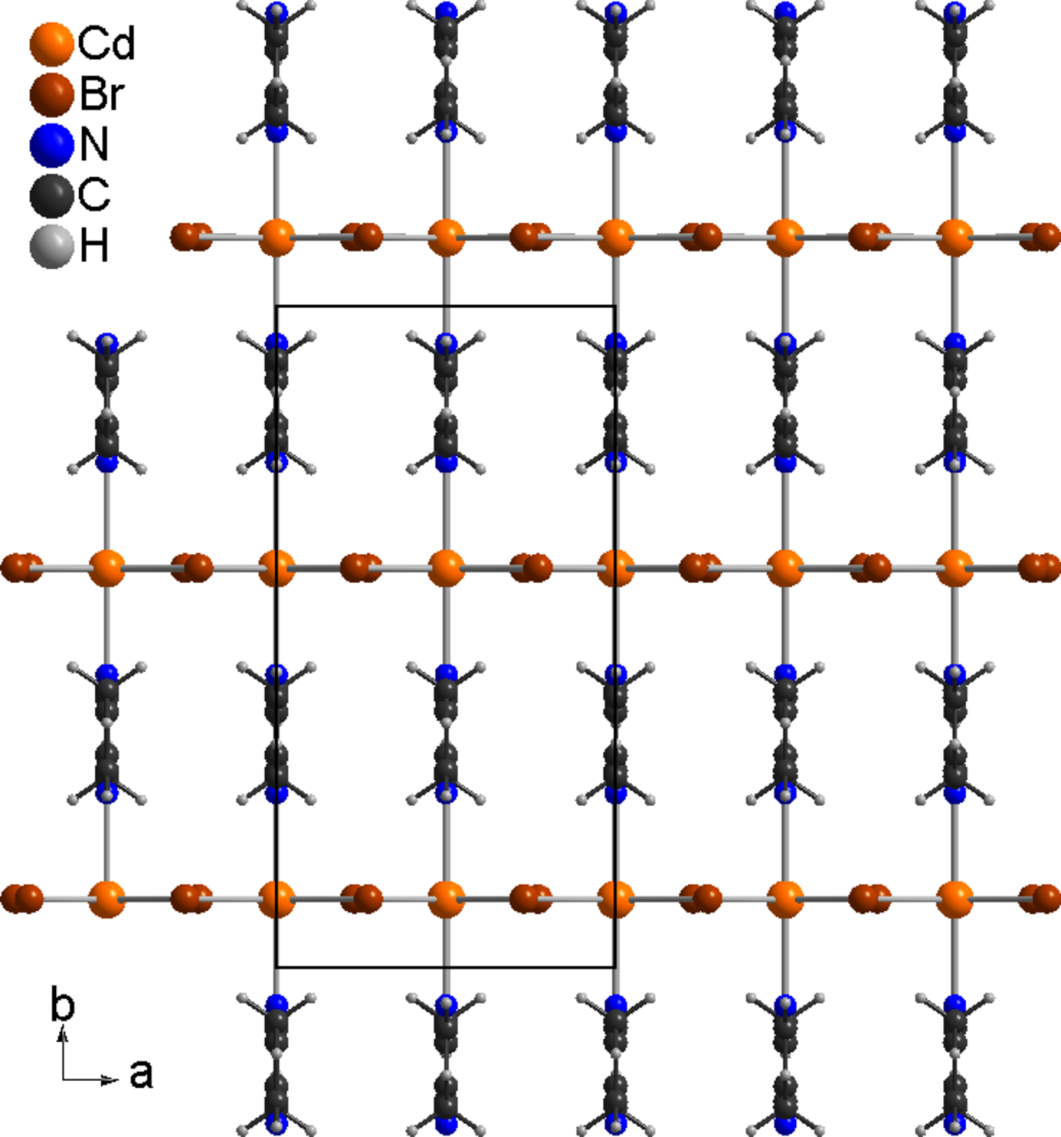
Crystal structure of (**I**) with view along the crystallographic *c*-axis direction.

The asymmetric unit of (**I**) consists of one Cd cation and one 2,5-di­methyl­pyrazine ligand that are located on a crystallographic mirror plane as well as one bromide anion that occupies a general position (Fig. 1 ▸). The Cd cations are octa­hedrally coordinated by four bromide anions that are located in the basal plane and two N-bonded 2,5-di­methyl­pyrazine ligands in the axial positions. The N—Cd—N and N—Cd—Br angles are close to the ideal values,whereas the Br—Cd—Br angles are significantly different from 90°, which shows that the octa­hedra are significantly distorted (Table 1 ▸). The cadmium cations are linked into chains *via* pairs of μ-1,1-bridging bromide anions that propagate in the crystallographic *a*-axis direction, which means that neighboring octa­hedra share common edges (Fig. 2 ▸). These chains are further linked into layers lying parallel to (001) by the bridging 2,5-di­methyl­pyrazine ligands (Fig. 3 ▸). It is noted that this topology is well known from Cd*X*_2_ compounds with pyrazine derivatives such as Cd*X*_2_(pyrazine) (*X* = Cl, Br, I) (Bailey & Pennington, 1997 ▸; Pickardt & Staub, 1997 ▸). It is also noted that the crystal structure of the title compound is completely different from the iodide analogue CdI_2_(2,5-di­methyl­pyrazine) already reported in the literature (EHEQUG; Rogers, 2020 ▸).

## 3. Supra­molecular features

**Figure 4:**
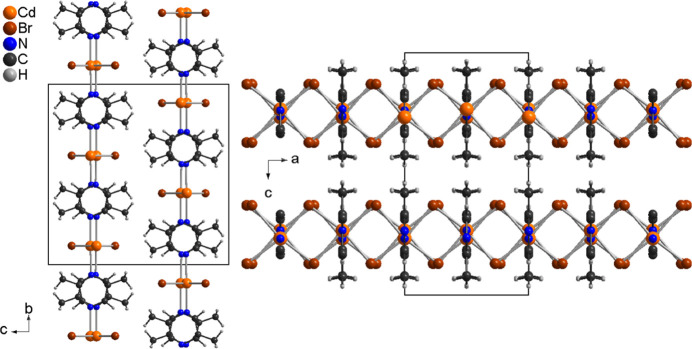
Crystal structure of (**I**) with view along the crystallographic *a*- (left) and *b*-axis (right) directions.

The layers in (**I**) are stacked in the crystallographic *c*-axis direction such that the methyl groups of neighboring layers point towards each along the *a*-axis direction or that they are shifted relative to each other along the *b*-axis direction so that the methyl groups are opposite to the bromide anions (Fig. 4 ▸). In the crystal structure of (**I**) a number of inter­molecular C—H⋯Br contacts are observed but for all of them the H⋯Br distances are very long and the C—H⋯Br angles are far from linear, indicating that these are only very weak inter­actions (Table 2 ▸).

## 4. Database survey

As mentioned in the *Chemical context* section, only one cadmium halide compound with 2,5-di­methyl­pyrazine is reported as a private communication in the CCDC database [CSD Version 5.43, September 2024 (Groom *et al.*, 2016 ▸), search with CONQUEST (Bruno *et al.*, 2002 ▸)]. However, a number of compounds with other twofold positively charged transition-metal cations, halide anions and 2,5-di­methyl­pyrazine are known. This include the two isotypic compounds *M*Br_2_(2,5-di­methyl­pyrazine), in which the metal cations are square-planar coordinated by two bromide anions and two 2,5-di­methyl­pyrazine ligands and linked into chains by the neutral coligands [*M* = Ni (BRMPYN; Ayres *et al.*, 1964 ▸) and *M* = Cu (DOVNUY; Butcher *et al.*, 2009 ▸)]. The same structure is also observed in CuCl_2_(2,5-di­methyl­pyrazine) (RAZYEX; Awwadi *et al.*, 2005 ▸), but this compound is not isotypic to the bromide compounds mentioned before.

Several compounds are reported with Zn^II^ in which the Zn^II^ cations are tetra­hedrally coordinated, including Zn*X*_2_(2,5-di­methyl­pyrazine) in which the Zn^II^ cations are linked into chains by the 2,5-di­methyl­pyrazine ligands (*X* = Cl, DOPYAJ, *X* = Br, DOPYIR, *X* = I, DOPZAK; Wriedt *et al.*, 2009 ▸). In (Zn*X*_2_)_2_(2,5-di­methyl­pyrazine)_3_ dinuclear complexes are formed in which the 2,5-di­methyl­pyrazine acts as bridging and terminal ligands (*X* = Cl, DOPYEN, *X* = Br, DOPYOX; Wriedt *et al.*, 2009 ▸). In ZnBr_2_(2,5-di­methyl­pyrazine)_2_-2,5-di­methyl­pyrazine solvate, discrete complexes are observed (DOPYUD; Wriedt *et al.*, 2009 ▸). Discrete complexes are also observed in ZnI_2_(2,5-di­methyl­pyrazine)_2_ (DOPZEO; Wriedt *et al.*, 2009 ▸). Additional compounds are reported with Cu^II^ cations, including CuBr_2_(2,5-di­methyl­pyrazine)(aceto­nitrile), in which the Cu^II^ cations are fivefold coordinated by two chloride anions, one aceto­nitrile ligand and two bridging 2,5-di­methyl­pyrazine ligands that link the cations into chains (MEVRAG; Näther & Greve, 2001 ▸).

Finally, two compounds with the composition (Hg*X*_2_)_2_(2,5-di­methyl­pyrazine) (*X* = Cl, QUMVIE, *X* = Br, QUMTUO; Mahmoudi & Morsali, 2009 ▸) are also reported, and show a topology similar to that of the title compound.

## 5. Additional investigations

**Figure 5:**
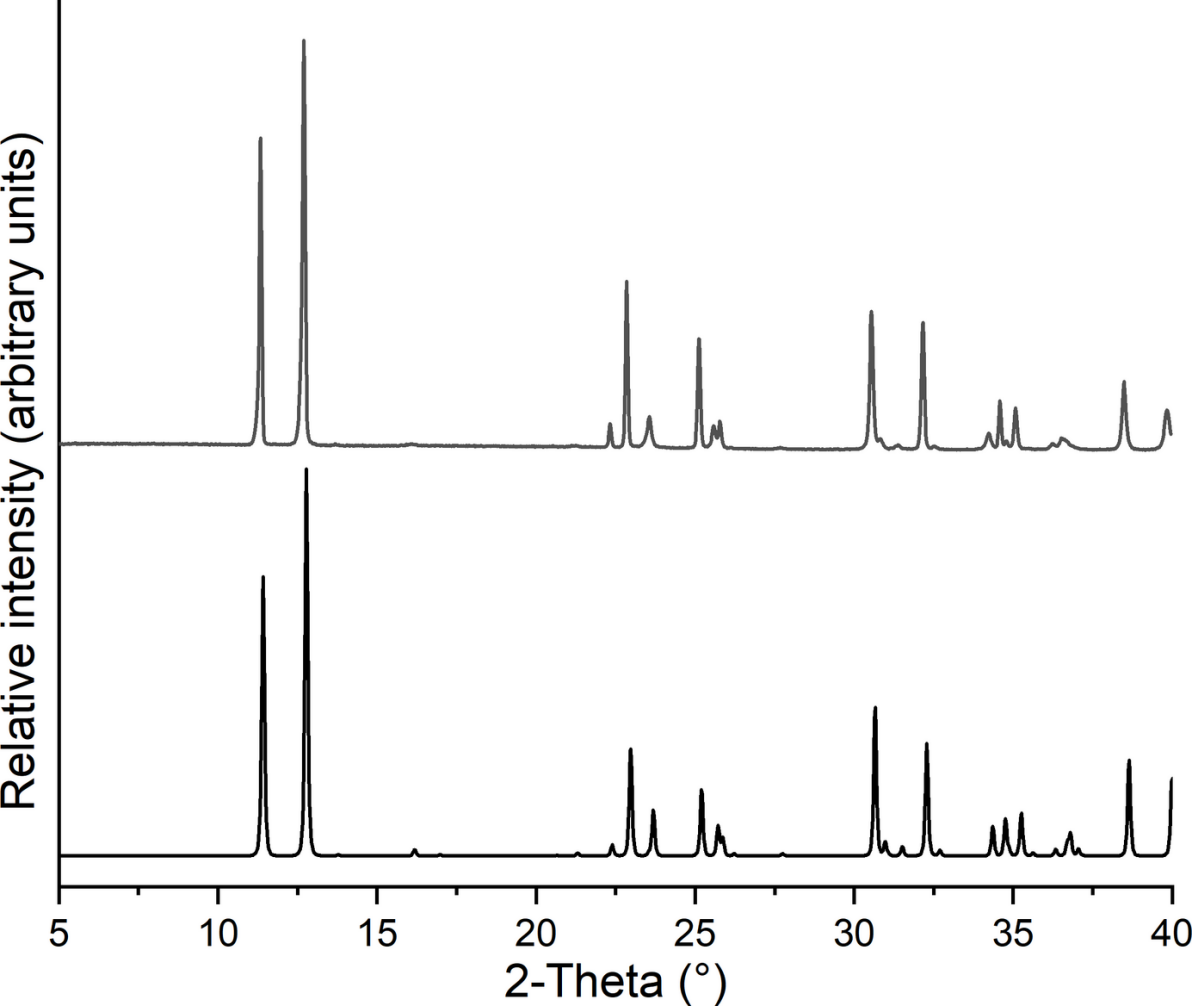
Experimental (top) and calculated X-ray powder pattern (bottom) for (**I**).

Comparison of the the experimental X-ray powder pattern of the sample with that calculated for the title compound from single-crystal data shows that a pure crystalline phase has been obtained (Fig. 5 ▸).

**Figure 6:**
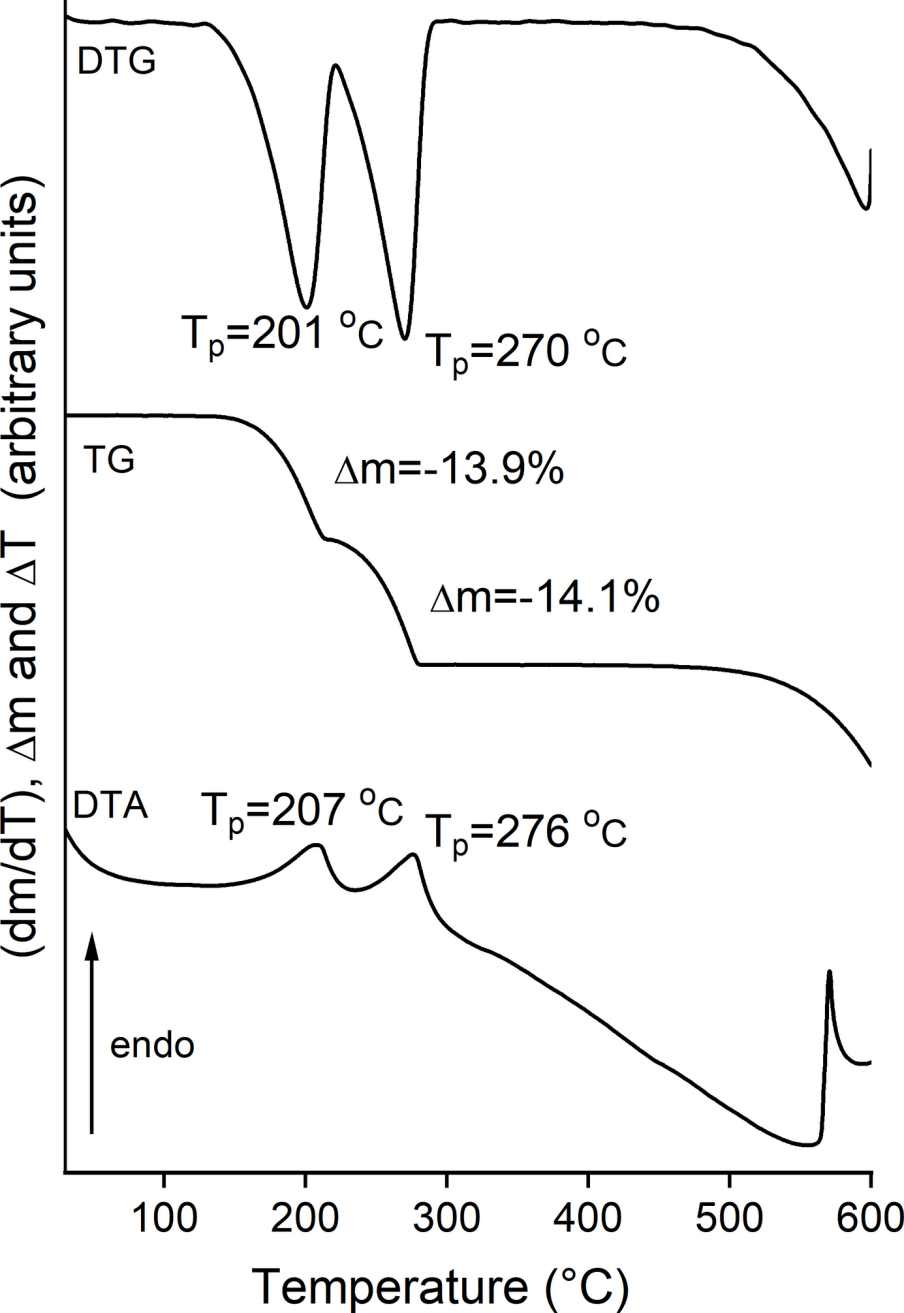
DTG, TG and DTA curves for (**I**) measured with a heating rate of 4°C min^−1^. The mass loss is given in % and the peak temperature in °C.

Thermogravimetry and differential thermoanalysis (TG-DTA) measurements reveal that (**I**) decomposes in two steps that are accompanied with endothermic events in the DTA curve at peak temperatures of 207 and 276°C (Fig. 6 ▸). The experimental mass losses of the first and second thermogravimetric step of 13.9 and 14.1% are in good agreement with those calculated for the removal of a half 2,5-di­methyl­pyrazine ligand in each step (Δ*m*_calc._ = 14.2%). Therefore, one can assume that after the first mass loss a compound with the composition (CdBr_2_)_2_(2,5-di­methyl­pyrazine) is formed, which decomposes into CdBr_2_ upon further heating.

**Figure 7:**
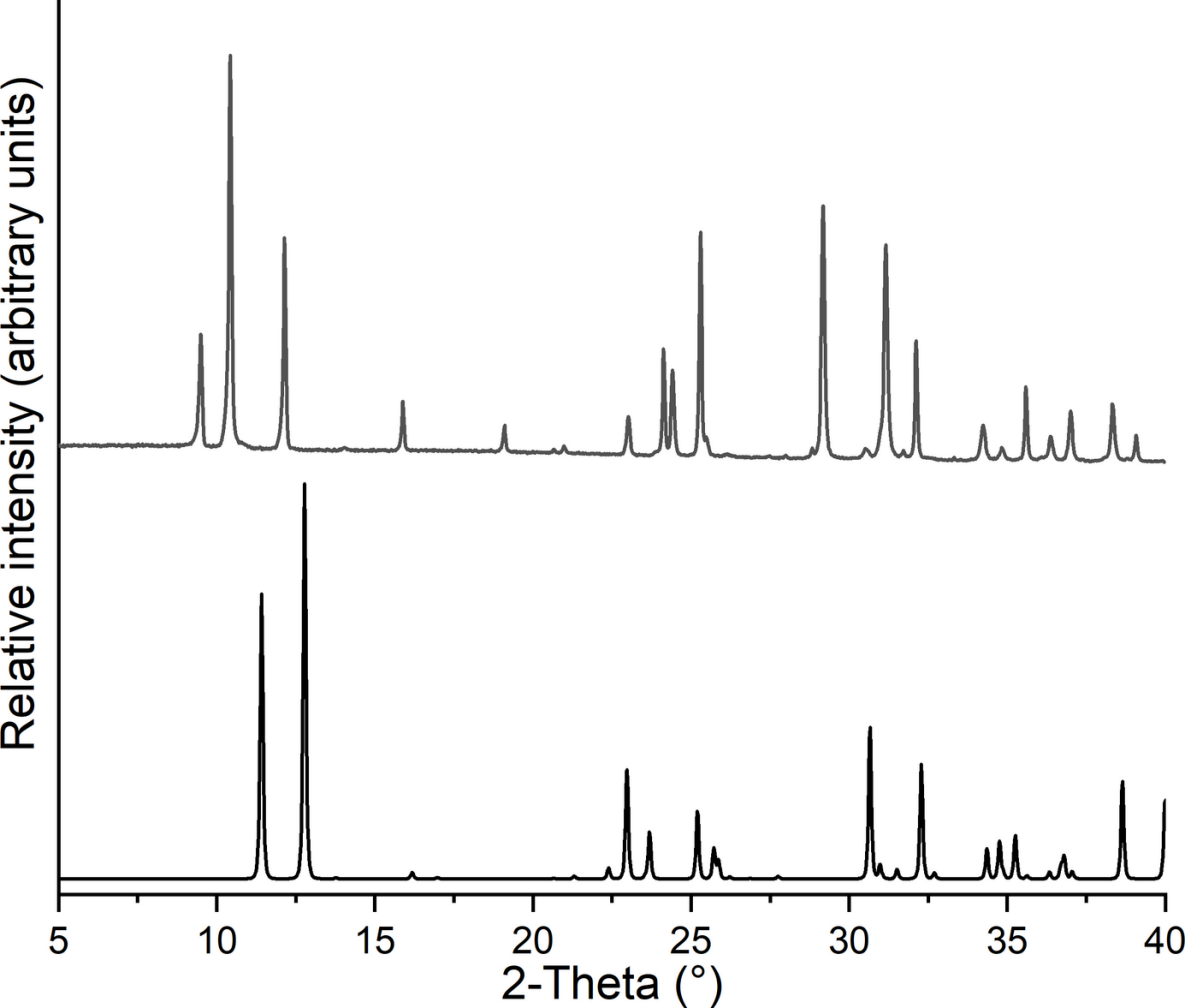
Experimental X-ray powder pattern of the residue obtained after the first mass loss in a TG measurement for (**I**) (top) and calculated powder pattern for (**I**) (bottom).

To prove that a new crystalline phase had formed, the residue obtained after the first mass loss was investigated by powder X-ray diffraction, which shows that a compound with very good crystallinity was obtained with a powder pattern completely different from that of the title compound (Fig. 7 ▸). Unfortunately, indexing of this powder pattern failed, so that no structural information is available. However, it can be assumed that a more condensed CdBr_2_ network formed.

## 6. Synthesis and crystallization

CdBr_2_ and 2,5-di­methyl­pyrazine were purchased from Sigma-Aldrich. 136.1 mg (0.5 mmol) of CdBr_2_ and 54.1 mg of (0.5 mmol) 2,5-di­methyl­pyrazine were reacted in 2 ml of water for 2 d at 353 K, which led to the formation of colorless crystals suitable for single crystal X-ray diffraction.

The PXRD measurements were performed with Cu *K*α_1_ radiation (λ = 1.540598 Å) using a Stoe Transmission Powder Diffraction System (STADI P) equipped with a MYTHEN 1K detector and a Johansson-type Ge(111) monochromator. Thermogravimetry and differential thermoanalysis (TG-DTA) experiments were performed in a dynamic nitro­gen atmosphere in Al_2_O_3_ crucibles with 8°C min^−1^ using a STA-PT 1000 thermobalance from Linseis. The TG-DTA instrument was calibrated using standard reference materials.

## 7. Refinement

Crystal data, data collection and structure refinement details are summarized in Table 3 ▸. The C—H hydrogen atoms were positioned with idealized geometry (methyl H atoms allowed to rotate but not to tip) and were refined isotropically with *U*_iso_(H) = 1.2*U*_eq_(C) (1.5 for methyl H atoms).

## Supplementary Material

Crystal structure: contains datablock(s) I, global. DOI: 10.1107/S2056989024011824/hb8115sup1.cif

Structure factors: contains datablock(s) I. DOI: 10.1107/S2056989024011824/hb8115Isup2.hkl

CCDC reference: 2407788

Additional supporting information:  crystallographic information; 3D view; checkCIF report

## Figures and Tables

**Table 1:** Selected geometric parameters (Å, °)

Cd1—Br1	2.6869 (4)	Cd1—N1	2.482 (5)
Cd1—Br1^i^	2.7893 (4)	Cd1—N2^ii^	2.494 (5)
			
Br1—Cd1—Br1^iii^	106.18 (2)	N1—Cd1—Br1	91.20 (6)
Br1—Cd1—Br1^iv^	86.222 (11)	N1—Cd1—N2^ii^	178.93 (15)
Br1^i^—Cd1—Br1^iv^	81.37 (2)	N2^ii^—Cd1—Br1^iii^	89.45 (6)
Br1^iii^—Cd1—Br1^iv^	167.574 (18)	N2^ii^—Cd1—Br1^iv^	89.94 (8)
N1—Cd1—Br1^iv^	89.25 (8)	Cd1—Br1—Cd1^v^	93.774 (11)

Symmetry codes: (i) 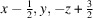; (ii) 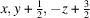; (iii) 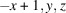; (iv) 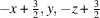; (v) 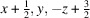.

**Table 2:** Hydrogen-bond geometry (Å, °)

*D*—H⋯*A*	*D*—H	H⋯*A*	*D*⋯*A*	*D*—H⋯*A*
C2—H2⋯Br1^vi^	0.95	3.02	3.624 (5)	123
C2—H2⋯Br1^vii^	0.95	3.02	3.624 (5)	123
C4—H4⋯Br1^i^	0.95	3.04	3.622 (5)	121
C4—H4⋯Br1^iv^	0.95	3.04	3.622 (5)	121
C5—H5*B*⋯Br1	0.98	2.83	3.658 (5)	142
C5—H5*C*⋯Br1^iii^	0.98	2.83	3.658 (5)	142
C6—H6*B*⋯Br1^viii^	0.98	2.86	3.674 (5)	141
C6—H6*C*⋯Br1^ix^	0.98	2.86	3.674 (5)	141

Symmetry codes: (i) 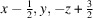; (iii) 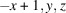; (iv) 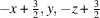; (vi) 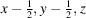; (vii) 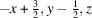; (viii) 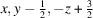; (ix) 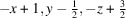.

**Table 3:** Experimental details

Crystal data	
Chemical formula	[CdBr_2_(C_6_H_8_N_2_)]
*M*_r_	380.36
Crystal system, space group	Orthorhombic, *Cmce*
Temperature (K)	170
*a*, *b*, *c* (Å)	7.9334 (2), 15.4735 (6), 15.4898 (6)
*V* (Å^3^)	1901.49 (11)
*Z*	8
Radiation type	Mo *K*α
μ (mm^−1^)	10.64
Crystal size (mm)	0.14 × 0.11 × 0.07

Data collection	
Diffractometer	Stoe *IPDS2*
Absorption correction	Numerical
*T*_min_, *T*_max_	0.193, 0.276
No. of measured, independent and observed [*I* > 2σ(*I*)] reflections	14714, 1238, 1082
*R*_int_	0.039
(sin θ/λ)_max_ (Å^−1^)	0.661

Refinement	
*R*[*F*^2^ > 2σ(*F*^2^)], *wR*(*F*^2^), *S*	0.033, 0.088, 1.13
No. of reflections	1238
No. of parameters	64
H-atom treatment	H-atom parameters constrained
Δρ_max_, Δρ_min_ (e Å^−3^)	1.99, −0.84

Computer programs: *X-AREA* (Stoe, 2008 ▸), *SHELXT2014/4* (Sheldrick, 2015*a* ▸), *SHELXL2016/6* (Sheldrick, 2015*b* ▸), *DIAMOND* (Brandenburg, 1999 ▸), *XP* in *SHELXTL-PC* (Sheldrick, 2008 ▸) and *publCIF* (Westrip, 2010 ▸).

## supplementary crystallographic information

### Poly[di-µ-bromido-(µ-2,5-dimethylpyrazine)cadmium(II)] Crystal data

**Table:**

[CdBr_2_(C_6_H_8_N_2_)]	*D*_x_ = 2.657 Mg m^−^^3^
*M**_r_* = 380.36	Mo *K*α radiation, λ = 0.71073 Å
Orthorhombic, *C**m**c**e*	Cell parameters from 15283 reflections
*a* = 7.9334 (2) Å	θ = 5.3–57.8°
*b* = 15.4735 (6) Å	µ = 10.64 mm^−^^1^
*c* = 15.4898 (6) Å	*T* = 170 K
*V* = 1901.49 (11) Å^3^	Block, colorless
*Z* = 8	0.14 × 0.11 × 0.07 mm
*F*(000) = 1408	

### Poly[di-µ-bromido-(µ-2,5-dimethylpyrazine)cadmium(II)] Data collection

**Table:**

Stoe IPDS-2 diffractometer	1082 reflections with *I* > 2σ(*I*)
ω scans	*R*_int_ = 0.039
Absorption correction: numerical	θ_max_ = 28.0°, θ_min_ = 2.6°
*T*_min_ = 0.193, *T*_max_ = 0.276	*h* = −10→10
14714 measured reflections	*k* = −20→20
1238 independent reflections	*l* = −20→20

### Poly[di-µ-bromido-(µ-2,5-dimethylpyrazine)cadmium(II)] Refinement

**Table:**

Refinement on *F*^2^	0 restraints
Least-squares matrix: full	Hydrogen site location: inferred from neighbouring sites
*R*[*F*^2^ > 2σ(*F*^2^)] = 0.033	H-atom parameters constrained
*wR*(*F*^2^) = 0.088	*w* = 1/[σ^2^(*F*_o_^2^) + (0.0592*P*)^2^] where *P* = (*F*_o_^2^ + 2*F*_c_^2^)/3
*S* = 1.13	(Δ/σ)_max_ = 0.001
1238 reflections	Δρ_max_ = 1.99 e Å^−^^3^
64 parameters	Δρ_min_ = −0.84 e Å^−^^3^

### Poly[di-µ-bromido-(µ-2,5-dimethylpyrazine)cadmium(II)] Special details

**Table:**

Geometry. All esds (except the esd in the dihedral angle between two l.s. planes) are estimated using the full covariance matrix. The cell esds are taken into account individually in the estimation of esds in distances, angles and torsion angles; correlations between esds in cell parameters are only used when they are defined by crystal symmetry. An approximate (isotropic) treatment of cell esds is used for estimating esds involving l.s. planes.

### Poly[di-µ-bromido-(µ-2,5-dimethylpyrazine)cadmium(II)] Fractional atomic coordinates and isotropic or equivalent isotropic displacement parameters (Å^2^)

**Table:**

	*x*	*y*	*z*	*U*_iso_*/*U*_eq_	Occ. (<1)
Cd1	0.500000	0.60333 (2)	0.76619 (2)	0.02282 (15)	
Br1	0.77081 (5)	0.60437 (3)	0.87036 (2)	0.02692 (15)	
N1	0.500000	0.4430 (3)	0.7622 (3)	0.0250 (9)	
C1	0.500000	0.3874 (3)	0.8292 (4)	0.0275 (11)	
C2	0.500000	0.2984 (4)	0.8119 (4)	0.0294 (12)	
H2	0.500000	0.259776	0.859575	0.035*	
N2	0.500000	0.2645 (3)	0.7328 (3)	0.0263 (10)	
C3	0.500000	0.3214 (4)	0.6659 (3)	0.0267 (11)	
C4	0.500000	0.4088 (4)	0.6818 (4)	0.0264 (11)	
H4	0.500000	0.447104	0.633895	0.032*	
C5	0.500000	0.4195 (4)	0.9195 (3)	0.0350 (14)	
H5A	0.500000	0.370280	0.959327	0.052*	
H5B	0.600860	0.454687	0.929396	0.052*	0.5
H5C	0.399140	0.454687	0.929396	0.052*	0.5
C6	0.500000	0.2890 (4)	0.5746 (4)	0.0350 (14)	
H6A	0.500000	0.338159	0.534803	0.052*	
H6B	0.600860	0.253755	0.564744	0.052*	0.5
H6C	0.399140	0.253755	0.564744	0.052*	0.5

### Poly[di-µ-bromido-(µ-2,5-dimethylpyrazine)cadmium(II)] Atomic displacement parameters (Å^2^)

**Table:**

	*U*^11^	*U*^22^	*U*^33^	*U*^12^	*U*^13^	*U*^23^
Cd1	0.0223 (2)	0.0213 (2)	0.0248 (2)	0.000	0.000	0.00021 (14)
Br1	0.0231 (2)	0.0340 (3)	0.0237 (2)	−0.00048 (15)	0.00010 (13)	−0.00062 (14)
N1	0.031 (2)	0.022 (2)	0.022 (2)	0.000	0.000	0.0015 (16)
C1	0.031 (3)	0.025 (3)	0.026 (3)	0.000	0.000	0.0040 (19)
C2	0.036 (3)	0.026 (3)	0.026 (2)	0.000	0.000	0.003 (2)
N2	0.030 (2)	0.021 (2)	0.028 (2)	0.000	0.000	−0.0030 (17)
C3	0.029 (3)	0.025 (3)	0.026 (2)	0.000	0.000	0.002 (2)
C4	0.028 (3)	0.028 (3)	0.023 (2)	0.000	0.000	0.0009 (19)
C5	0.057 (4)	0.026 (3)	0.022 (2)	0.000	0.000	−0.001 (2)
C6	0.056 (4)	0.023 (3)	0.026 (2)	0.000	0.000	−0.004 (2)

### Poly[di-µ-bromido-(µ-2,5-dimethylpyrazine)cadmium(II)] Geometric parameters (Å, º)

**Table:**

Cd1—Br1	2.6869 (4)	C2—N2	1.332 (7)
Cd1—Br1^i^	2.7893 (4)	N2—C3	1.360 (7)
Cd1—Br1^ii^	2.6869 (4)	C3—C4	1.374 (8)
Cd1—Br1^iii^	2.7893 (4)	C3—C6	1.500 (7)
Cd1—N1	2.482 (5)	C4—H4	0.9500
Cd1—N2^iv^	2.494 (5)	C5—H5A	0.9800
N1—C1	1.347 (7)	C5—H5B	0.9800
N1—C4	1.353 (7)	C5—H5C	0.9800
C1—C2	1.404 (8)	C6—H6A	0.9800
C1—C5	1.484 (8)	C6—H6B	0.9800
C2—H2	0.9500	C6—H6C	0.9800
			
Br1—Cd1—Br1^ii^	106.18 (2)	N2—C2—C1	124.2 (5)
Br1—Cd1—Br1^iii^	86.222 (11)	N2—C2—H2	117.9
Br1^ii^—Cd1—Br1^i^	86.222 (11)	C2—N2—Cd1^vi^	112.8 (4)
Br1^i^—Cd1—Br1^iii^	81.37 (2)	C2—N2—C3	116.5 (5)
Br1^ii^—Cd1—Br1^iii^	167.574 (18)	C3—N2—Cd1^vi^	130.7 (3)
Br1—Cd1—Br1^i^	167.574 (18)	N2—C3—C4	120.0 (5)
N1—Cd1—Br1^iii^	89.25 (8)	N2—C3—C6	120.1 (5)
N1—Cd1—Br1^ii^	91.20 (6)	C4—C3—C6	119.9 (5)
N1—Cd1—Br1	91.20 (6)	N1—C4—C3	123.4 (5)
N1—Cd1—Br1^i^	89.25 (8)	N1—C4—H4	118.3
N1—Cd1—N2^iv^	178.93 (15)	C3—C4—H4	118.3
N2^iv^—Cd1—Br1^ii^	89.45 (6)	C1—C5—H5A	109.5
N2^iv^—Cd1—Br1	89.45 (6)	C1—C5—H5B	109.5
N2^iv^—Cd1—Br1^i^	89.94 (8)	C1—C5—H5C	109.5
N2^iv^—Cd1—Br1^iii^	89.94 (8)	H5A—C5—H5B	109.5
Cd1—Br1—Cd1^v^	93.774 (11)	H5A—C5—H5C	109.5
C1—N1—Cd1	128.2 (4)	H5B—C5—H5C	109.5
C1—N1—C4	117.3 (5)	C3—C6—H6A	109.5
C4—N1—Cd1	114.4 (4)	C3—C6—H6B	109.5
N1—C1—C2	118.6 (5)	C3—C6—H6C	109.5
N1—C1—C5	120.8 (5)	H6A—C6—H6B	109.5
C2—C1—C5	120.6 (5)	H6A—C6—H6C	109.5
C1—C2—H2	117.9	H6B—C6—H6C	109.5
			
Cd1—N1—C1—C2	180.0	C1—C2—N2—C3	0.0
Cd1—N1—C1—C5	0.0	C2—N2—C3—C4	0.0
Cd1—N1—C4—C3	180.0	C2—N2—C3—C6	180.0
Cd1^vi^—N2—C3—C4	180.0	N2—C3—C4—N1	0.0
Cd1^vi^—N2—C3—C6	0.0	C4—N1—C1—C2	0.0
N1—C1—C2—N2	0.0	C4—N1—C1—C5	180.0
C1—N1—C4—C3	0.0	C5—C1—C2—N2	180.0
C1—C2—N2—Cd1^vi^	180.0	C6—C3—C4—N1	180.0

Symmetry codes: (i) *x*−1/2, *y*, −*z*+3/2; (ii) −*x*+1, *y*, *z*; (iii) −*x*+3/2, *y*, −*z*+3/2; (iv) *x*, *y*+1/2, −*z*+3/2; (v) *x*+1/2, *y*, −*z*+3/2; (vi) *x*, *y*−1/2, −*z*+3/2.

### Poly[di-µ-bromido-(µ-2,5-dimethylpyrazine)cadmium(II)] Hydrogen-bond geometry (Å, º)

**Table:**

*D*—H···*A*	*D*—H	H···*A*	*D*···*A*	*D*—H···*A*
C2—H2···Br1^vii^	0.95	3.02	3.624 (5)	123
C2—H2···Br1^viii^	0.95	3.02	3.624 (5)	123
C4—H4···Br1^i^	0.95	3.04	3.622 (5)	121
C4—H4···Br1^iii^	0.95	3.04	3.622 (5)	121
C5—H5*B*···Br1	0.98	2.83	3.658 (5)	142
C5—H5*C*···Br1^ii^	0.98	2.83	3.658 (5)	142
C6—H6*B*···Br1^vi^	0.98	2.86	3.674 (5)	141
C6—H6*C*···Br1^ix^	0.98	2.86	3.674 (5)	141

Symmetry codes: (i) *x*−1/2, *y*, −*z*+3/2; (ii) −*x*+1, *y*, *z*; (iii) −*x*+3/2, *y*, −*z*+3/2; (vi) *x*, *y*−1/2, −*z*+3/2; (vii) *x*−1/2, *y*−1/2, *z*; (viii) −*x*+3/2, *y*−1/2, *z*; (ix) −*x*+1, *y*−1/2, −*z*+3/2.
